# Prevalence and Clinical Profile of Drug Eruptions among Antiretroviral Therapy-Exposed HIV Infected People in Yaoundé, Cameroon

**DOI:** 10.1155/2017/6216193

**Published:** 2017-06-28

**Authors:** Emmanuel Armand Kouotou, Jobert Richie Nansseu, Vanessa Nancy Ngono, Sandra A. Tatah, Anne Cecile Zoung-Kanyi Bissek, Elie Claude Ndjitoyap Ndam

**Affiliations:** ^1^Biyem-Assi District Hospital, Yaoundé, Cameroon; ^2^Yaoundé University Teaching Hospital, Yaoundé, Cameroon; ^3^Faculty of Medicine and Biomedical Sciences, University of Yaoundé I, Yaoundé, Cameroon

## Abstract

**Background:**

Prevalence and incidence of drug eruptions vary around the world and are influenced by some key factors including HIV infection.

**Objective:**

This study aimed to find the peculiarities of drug eruptions in people living with HIV (PLHIV) and on antiretroviral therapy (ART).

**Methods:**

This was a retrospective cross-sectional study including ART-taking PLHIV, aged 15+ years, followed up between January 2010 and December 2014 at the day-care unit of the Yaoundé Central Hospital, and who presented with drug eruptions after ART initiation.

**Results:**

Of 6,829 ART-experiencing PLHIV, 41 presented with drug eruptions, giving a prevalence of 0.6%. The M/F sex ratio equaled 0.17. The mean age was 41.07 ± 11.36 years. Benign drug eruptions accounted for 83.3%. Milder forms were essentially maculopapular exanthema (36.6%), fixed pigmented erythema (7.3%), and urticaria (4.9%). Severe forms were represented by multiform erythema (4.9%), toxic epidermal necrolysis (2.4%), and drug hypersensitivity syndrome (2.4%). The Zidovudine + Lamivudine + Efavirenz ART-protocol was received by 48.8% of patients and 69% of patients were receiving Cotrimoxazole prophylaxis. Nevirapine, Efavirenz, Zidovudine, and Cotrimoxazole were suspected as the potential causes in 43.7%, 4.8%, 2.4%, and 26.8% of cases, respectively.

**Conclusion:**

Drug eruptions seem infrequent among ART-exposed HIV infected adult Cameroonians.

## 1. Introduction

Drug eruptions are mucocutaneous complications due to administration of enteral, intravenous, subcutaneous, intramuscular, topical, or mucosal drugs [1, 2]. They are at the forefront of allergic or idiosyncratic iatrogenic incidents [3]. Although their pathophysiology is poorly understood, immunoallergic and pharmacological mechanisms have been suggested [2]. Clinical manifestations of drug eruptions are not very specific and sometimes clinically indistinguishable from other skin disorders [3]. The diagnosis of drug eruptions is a presumptive diagnosis based on a body of evidence, certainty being exceptional [1]. In practice, the diagnosis is bound to a particular diagnostic approach: the drug accountability, which is based on semiotic and chronological criteria [1, 4].

Milder forms of these skin manifestations are the most common, mainly represented by the maculopapular exanthema. They are usually all spontaneously favorable, not requiring discontinuation of treatment in 50% of cases [1, 5]. Their usual complications are unsightly pigmented scars [4]. However, severe forms, predominantly represented by the Drug Reaction with Eosinophilia and Systemic Symptoms (DRESS) syndrome, Stevens-Johnson syndrome, toxic epidermal necrolysis, and acute generalized pustular exanthematous pustulosis, can alter the functional prognosis or even become life threatening, with a mortality rate of about 30% in the West and around 70% in Africa [6, 7].

Drug eruptions are 20 to 100 times more common in people living with HIV (PLHIV) than in the general population. Sometimes they constitute the mode of HIV revelation [8]. Occurrence of severe forms of drug eruptions is more likely to happen during HIV infection [5]. This increasing trend for PLHIV to develop drug eruptions has been associated with the use of antiretroviral therapy (ART). Indeed, Amoussou et al. have shown the involvement of all classes of ART regimen in the occurrence of maculopapular exanthema and bullous forms of drug eruptions, especially the nonnucleoside reverse transcriptase inhibitors (NNRTIs) such as Nevirapine [9].

In sub-Saharan Africa, the epicenter of HIV infection, the incidence of drug eruptions is higher due to the large proportion of PLHIV. The prevalence of drug eruptions in PLHIV varies between 2 and 11.3% [10, 11]. In Cameroon specifically, in a study on mucocutaneous disorders in the course of HIV infection, Lando et al. found a prevalence of 2.1% in 2003 [12]. Since then, no other study had focused on drug eruptions in Cameroonian PLHIV. Hence, we conducted the present study to determine the prevalence of drug eruptions in ART-exposed PLHIV and describe the clinical context of occurrence, the clinical forms observed, ongoing treatment at the time of occurrence, and the therapeutic attitudes adopted.

## 2. Materials and Methods

### 2.1. Study Design and Setting

We conducted a retrospective study. Data were collected from January to May 2015 at the day-care unit of the Yaoundé Central Hospital. This is a treatment centre receiving and supporting PLHIV irrespective of their place of origin. Currently, 40,000 patients are registered and regularly followed up, which is by far the largest cohort in Cameroon.

### 2.2. Study Population, Procedures, and Data Collection

Inclusion criteria comprised (i) being aged 15 and above; (ii) being a PLHIV, registered and followed up at the study site between January 2010 and December 2014; (iii) being on continuous ART with a clearly stated therapeutic protocol; (iv) having presented with a confirmed drug eruption after initiation of ART, this being diagnosed by a dermatologist; (v) having shown a skin reaction very likely linked to use of a drug with an established extrinsic accountability; (vi) having a computerized medical file and a physical medical record at the onset of drug eruption. We excluded any patient with a drug eruption that was not confirmed after extraction of data from physical medical records, as well as any unconfirmed case of drug reactions in the computerized medical record.

For data collection, we used computerized patients' medical records which are compiled in a database called “ESOPE” (Evaluation and Monitoring of Operational ESTHER programs) along with the physical medical records. ESOPE is a clinical, biological, and therapeutic monitoring software tool for patients infected with HIV and coinfections in the management units of adult Cameroonians living with HIV. This software aims to facilitate the monitoring of patients under treatment or not and to help in efficiently managing the hole cohort and monitoring activities. The ESOPE database of the day-care unit of the Yaoundé Central Hospital is complete, up-to-date, and put under the close supervision of the Department of Disease, Epidemics and Pandemics Control of the Ministry of Public Health, Cameroon.

The preliminary step was the selection of eligible patients from the ESOPE database. The first variable of interest was “ERUP_CUT” corresponding to “rash.” This variable includes 4 terms: Y = Yes persistent, N = No, X = Yes punctual, and P = missing. We retained the Y and X modalities for the selection of cases. The “PLANI-LIB” variable (“other reason for consultation”) and three interfaces of the ESOPE software (clinical, events, and customized variables) then allowed us to proceed with careful sorting of the type of skin reaction. After this second selection we retained patients with skin lesions related to ART, skin lesions with no clinical information, skin lesions associated with an unspecified drug allergy, and skin lesions clearly reported as drug eruptions.

Data extraction was completed by consulting the physical medical records of patients. With the physical record number contained in the computerized record, we found patients' files in the archives of the study site. Using a data collection sheet, we identified patients' demographics, data from the physical examination (skin and mucous lesions observed, presence or absence of functional signs, and organ involvement), the diagnosis, WHO HIV infection clinical stages, and conditions that justified administration of a drug and all other medications the patient was taking at drug eruption onset.

### 2.3. Ethical Considerations

Prior to implementing this study, we obtained an ethical clearance from the Ethical Review Board of the Faculty of Medicine and Biomedical Sciences of the University of Yaoundé I, Cameroon, as well as an authorization from the Directory of the Yaoundé Central Hospital. The retrospective nature of this study did not permit requests for participants' consent. However, we made sure to keep all the information gathered confidential.

## 3. Results

### 3.1. Sociodemographic Characteristics and Prevalence

**Figure 1:**
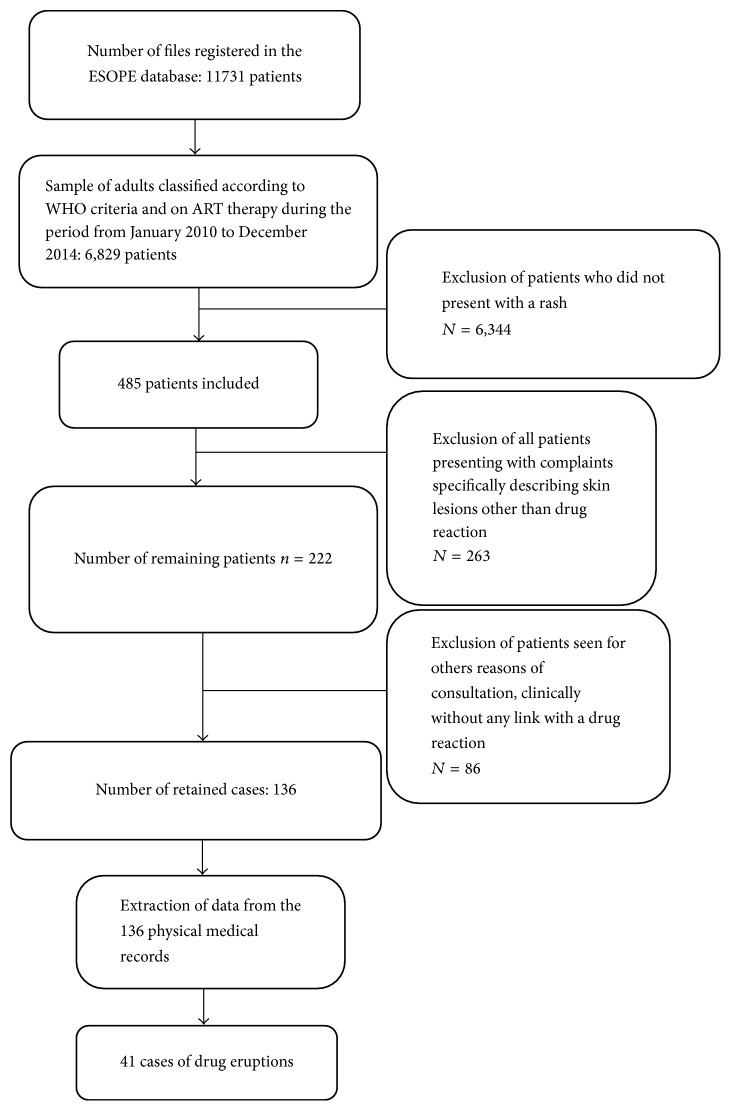
Algorithm describing the sampling procedure.

A total of 6,829 patients on ART and classified according to WHO criteria were recorded during the study period in the ESOPE database. After selection and meticulous sorting (Figure 1), 136 (2%) lesions probably corresponding to a drug eruption were identified. After extracting data from physical files, only 41 cases were found to be drug eruptions, thus giving a prevalence of 0.6% (Figure 1).

Our sample of 41 patients was predominantly made of female patients (34/41; 82.9%), with a M/F sex ratio of 0.17. The average age was 41.07 ± 11.36 years, with ages ranging from 22 to 70 years. Subjects between 30 and 38 years were those mostly represented (13/41; 31.7%).

### 3.2. Therapy at the Time of Onset of Drug Eruption and Subsequent Therapeutic Approach

Cotrimoxazole prophylaxis for opportunistic infections (toxoplasmosis, pneumocystis) was found in 61% (25/41) of patients. Administration of curative dose of Cotrimoxazole was found in one patient who presented with cerebral toxoplasmosis. A curative treatment with quinine was administered to a single case of malaria found in our sample. Another identified comorbidity was dysentery, treated with Mebendazole.

ART regimen combining lamivudine, Efavirenz, and Nevirapine was received by 48.8% (20/41) of patients. Protocols including “Tenofovir + Lamivudine + Efavirenz” and “Tenofovir + Lamivudine + Nevirapine” were received by 14.6% (6/41) of patients each. Nevirapine was found in the ART combination of 27/41 (65.9%) patients and Efavirenz was present in that of 14/41 (31.1%) patients.

### 3.3. Drug Eruptions Observed

Aside from the “probable drug eruptions,” benign forms of drug reactions accounted for 83.3% (20/24) and severe forms for 16.7% (4/24) of drug eruptions. The drug eruption mostly found was maculopapular exanthema (15/41; 36.6%). Three forms of severe drug eruptions were found including multiform erythema (2/41; 4.9%), toxic epidermal necrolysis (1/41; 2.4%), and DRESS syndrome (1/41; 2.4%) (Table 1).

### 3.4. Drugs Suspected in the Onset of Drug Eruption

Based on the extrinsic accountability, drugs involved in the onset of the drug eruption could be determined. Nevirapine was the drug mostly suspected in the occurrence of drug eruptions (18/41; 43.9%). It probably induced the majority of maculopapular exanthema (9/41; 22%). One case of fixed pigmented erythema was also noticed in a patient on Nevirapine. Nevirapine was suspected in the occurrence of two severe drug reactions including multiform erythema (1/41; 2.4%) and toxic epidermal necrolysis (1/41; 2.4%). In the “probable drug eruption” group, Nevirapine was strongly suspected in 8 patients (19.5%).

Cotrimoxazole followed Nevirapine with 11 cases (26.8%) of extrinsic accountability. Milder forms probably induced by Cotrimoxazole were maculopapular exanthema (2/41; 4.9%), fixed pigmented erythema (2/41; 4.9%), and urticaria (1/41; 2.4%). The only case of DRESS was also suspected to have been induced by Cotrimoxazole. Five “probable drug eruptions” were potentially attributable to Cotrimoxazole.

Efavirenz was likely implicated in the onset of multiform erythema (1/41; 2.4%). Additionally, a case of “probable drug eruption” was suspected to have been caused by Efavirenz. Meanwhile, Zidovudine probably induced an acute urticaria (2.4%).

The drug inducing eruptions was not specifically identified in 9/41 patients (22%). On one hand, in 3 cases of maculopapular exanthema and 4 cases of “probable drug eruptions,” implication of Cotrimoxazole or one of ART molecules was clearly not established. On the other hand, we found a case of maculopapular exanthema and a case of “probable drug eruption” in which the potentially attributable ART was not determined.

In all, antiretrovirals were clearly reported as potential inducing drugs in the occurrence of 23 cases of drug eruptions (56.1%, Table 2).

### 3.5. Subsequent Therapeutic Approach to Revert the Drug Eruptions

Antiretrovirals were suspected in causing 29 (70.7%) drug eruptions. The likely inducing ART molecule was discontinued and replaced in 20/29 (69%) of cases. For the 9 remaining cases, the suspected ART molecule was continued. A stop without substitution of Cotrimoxazole was done in 7/11 patients (63.6%) and 4/11 (36.4%) continued taking Cotrimoxazole.

## 4. Discussion

This study revealed a prevalence of 0.6% for drug eruptions among PLHIV and those taking ART. This prevalence shows that drug eruptions seem infrequent in PLHIV on ART in our environment, particularly in this Treatment Centre of Yaoundé. Our prevalence is lower than the values found in the African and Eastern literatures. Indeed, this was estimated at 2.8% by Salami et al. in Nigeria [10] and at 1.6% by Ananworanich et al. in Thailand [13]. Our prevalence can be explained by the fact that, in our study, patients included were aged 15 years and above and had to be on ART unlike the other studies. Also, they were all recruited at only one site.

We observed a female predominance with a M/F sex ratio of 0.17. This distribution is consistent with the majority of African studies on drug eruptions in PLHIV. Pitche et al. in 2005 determined the “female sex” as a major risk factor for onset of drug eruptions with Nevirapine use [14]. Similarly, in the study by Diop et al. in 2014 in Senegal, 85.7% of drug eruptions occurred in women [15]. This strong female predominance is due to the susceptibility of the female sex to drug hypersensitivity [16] as well as the feminization of the HIV infection in sub-Saharan Africa [17].

Among the clearly individualized drug eruptions, benign forms of drug eruptions were the most frequent (83.3%). These results are close to that from the literature [5, 9]. Among these individualized forms (24/41), maculopapular exanthema was the most common drug eruption in our study (36.6%). This trend was similarly observed in other studies on PLHIV [14, 15].

Severe forms represented 16.7% of our sample, confirming their lower frequency in the course of HIV infection [5]. These were toxic epidermal necrolysis, DRESS syndrome, and 2 cases of multiform erythema. Our results do concur with those from Diop et al. [15] who found that 25.7% of patients had severe forms of which 7% were toxic epidermal necrolysis and 2.9% the DRESS syndrome.

All cases of drug eruptions occurred in patients receiving first-line ART regimens. The protocol “Lamivudine + Zidovudine + Nevirapine” was received by 48.8% of patients and 65.8% of patients were taking a protocol containing Nevirapine. Diop et al. found similar data in the proportions of 68.6% and 78.6%, respectively [15]. Efavirenz was found in 34.1% of ART regimens. Our results suggest the involvement of ART in the occurrence of drug side effects, including drug eruptions. In Nigeria, Eluwa et al. in 2012 and Agu et al. in 2013 determined that drug eruptions, respectively, represented 18% and 11.4% of ART side effects [18, 19].

The three ART molecules likely to induce drug eruptions in our series were Nevirapine, Efavirenz, and Zidovudine. In 2007 in Mali, Kane et al. also identified these three as the molecules responsible for drug eruptions [11]. Our prevalence of drug eruptions probably induced by Nevirapine (43.1%) is lower than that found by Diop et al. (87.1%) [15]. The maculopapular exanthema was the most common drug eruption likely due to Nevirapine, which corroborates existing data [9, 13]. Efavirenz was suspected in the occurrence of 4.8% cases of drug eruptions, very different from the 20% reported by Ananworanich et al. in Thailand [13]. This is explained by the greater number of patients on Efavirenz-containing ART in their study. The proportion of drug eruptions induced by Efavirenz found in our study is consistent with the literature which is indicative of a lower involvement of Efavirenz in the occurrence of drug eruptions although belonging to the same family as Nevirapine [20].

Cotrimoxazole was suspected to have induced 26.8% of the observed drug eruptions, most often benign. Two cases of fixed drug eruptions likely induced by Cotrimoxazole were recorded in our study, as in the study by Saka et al. [21]. In 7 cases (17%), the incriminating drug (Cotrimoxazole or ART) was not determined. This would be an indicator of a weakness of the pharmacovigilance system in our context.

In the literature, several authors recommend continuation of treatment in more than 50% of cases when it is a benign drug eruption, both in PLHIV and in the general population [1, 5]. In our study, a substitution of the suspected molecule was made in 51.2% of cases of drug eruptions. Moreover, the drug in question was pursued in 31.8% of drug eruptions. This high frequency of drug substitution is contradictory to the high percentage of benign drug eruptions found in our study. This therapeutic attitude of the medical staff can be explained in this context by the fear of an evolution from a mild to a severe form with poor prognosis.

Some limitations to this work are worth noticing, including the size of our sample which consisted of only ART-taking PLHIV, aged 15 years and above. In addition, it was difficult to identify a single drug responsible for adverse reactions, especially in patients taking multiple drugs (ART, Cotrimoxazole, and other drugs). The retrospective design of our study could be another limit because we had multiple physical medical records with incomplete or improperly filled data. Nevertheless, we used a rigorous selection procedure to select and retain patients for inclusion in this study. The fact that our study was conducted at a single site makes it difficult to extrapolate our findings to the whole country. However, this centre contains the largest cohort of PLHIV in Cameroon, patients coming from Yaoundé and the surrounding towns.

## 5. Conclusion

This work has shown that drug eruptions seem infrequent among ART-taking PLHIV followed up at the Yaoundé Central Hospital, Cameroon, with a prevalence around 1%. These skin disorders seem more common among females and young adults. Benign drug eruptions are more often encountered; maculopapular exanthema is the drug eruption seen the most. The majority of drug eruptions are likely to be induced by ART and Cotrimoxazole. This study advocates the strengthening of the pharmacovigilance system in our setting. Moreover, further studies should be conducted to elucidate all the risk factors for drug eruptions occurrence in PLHIV residing in Cameroon, for their efficient prevention and proper management.

## Figures and Tables

**Table 1:** Different drug eruptions observed.

Drug eruptions observed	Number	Percentage (%)
Maculopapular exanthema	15	36.6
Probable drug eruption	17	41.4
Fixed pigmented erythema	3	7.3
Acute urticaria	2	4.9
Multiform erythema	2	4.9
Toxic epidermal necrolysis	1	2.4
DRESS syndrome	1	2.4
*Total*	*41*	*100*

**Table 2:** Drugs suspected depending on the nature of the drug eruption.

Type of drug eruption	Suspected drug					
	Specified ARV			CTX^*∗*^	ARV not specified	Doubt between CTX and ARV
	AZT	EFV	NVP			
Maculopapular exanthema	0	0	9	2	1	3
Urticaria	1	0	0	1	0	0
Fixed pigmented erythema	0	0	1	2	0	0
Toxic epidermal necrolysis	0	0	1	0	0	0
Probable drug eruptions	0	1	6	5	1	4
Erythema multiforme	0	1	1	0	0	0
DRESS syndrome	0	0	0	1	0	0
Total	1	2	18	11	2	7

^**∗**^CTX = Cotrimoxazole; AZT = Zidovudine; ARV = antiretroviral; EFV = Efavirenz; NVP = Nevirapine.
